# Use of Thrust Cervical Spinal Manipulative Therapy for Complicated Neck Pain: A Cross-Sectional Survey of Asia-Pacific Chiropractors

**DOI:** 10.7759/cureus.32441

**Published:** 2022-12-12

**Authors:** Eric C Chu, Robert J Trager, Wai T Lee

**Affiliations:** 1 New York Chiropractic and Physiotherapy Centre, New York Medical Group, Kowloon, HKG; 2 Chiropractic, Connor Whole Health, University Hospitals Cleveland Medical Center, Cleveland, USA; 3 College of Chiropractic, Logan University, Chesterfield, USA

**Keywords:** spinal fusion, arnold-chiari malformation, vertebral artery, contraindications, spinal manipulation, neck pain, chiropractic

## Abstract

Background

Chiropractors often use manual thrust cervical spinal manipulative therapy (thrust-cSMT) to treat musculoskeletal neck conditions. We hypothesized <50% of surveyed Asia-Pacific chiropractors would report using thrust-cSMT given potential contraindications, and secondarily explored predictors of thrust-cSMT use.

Materials and methods

We designed, validated, achieved sufficient reliability, and disseminated a survey to explore thrust-cSMT use. The survey queried chiropractors’ characteristics (e.g., years in practice, education level, time with patients, importance of subluxation), and use of thrust-cSMT for uncomplicated neck pain and vignettes describing vertebral artery disorders, Arnold-Chiari malformation, and anterior cervical discectomy and fusion (ACDF). We performed logistic regression for each vignette with thrust-cSMT as the dependent variable and chiropractor characteristics as covariates.

Results

There were 241 respondents, having 12.8±10.9 years in practice, representing >15 countries. Less than 50% of chiropractors reported the use of thrust-cSMT for each vignette, including vertebral artery insufficiency (14%) and stenosis (17%), Arnold-Chiari type I (18%) and type II (5%), C5/6 ACDF (39%) and C3-6 ACDF (27%). Regressions identified significant predictors of increased or decreased use of thrust-cSMT including time spent with new patients, focus on subluxation, degree, group practice environment, use of thrust-cSMT on a healthy patient, and hours reading scientific literature (P<.05 for each).

Conclusions

This study was the first to chiropractors’ use of thrust-cSMT for complicated neck pain and found that most Asia-Pacific chiropractors reported avoiding this treatment in the presence of a potential treatment contraindication. The use of thrust-cSMT in complicated neck pain may be related to practice characteristics. However, further research is needed to identify specific reasons why chiropractors use or avoid thrust-cSMT.

## Introduction

Chiropractors are portal-of-entry healthcare providers that frequently manage musculoskeletal conditions affecting the spine such as low back and neck pain [1]. Chiropractors often use cervical spinal manipulative therapy (cSMT) to treat neck pain involving a manual thrust/impulse [1,2]. A recent scoping review concluded that serious adverse events are rare in relation to thrust-cSMT [3]. In addition, another review suggested that these adverse events may be avoided when providers recognize contraindications to this therapy and do not use it [4]. However, it is unclear if chiropractors use thrust-cSMT when treating patients with underlying conditions that have been described as precautions or contraindications to this therapy.

Spinal manipulative therapies include a range of manual therapies directed to the joints of the spine [5]. However, these therapies can generally be divided into thrust (i.e., involving an impulse) and non-thrust techniques (i.e., low-grade mobilizations) [6,7]. Non-thrust techniques are generally recommended in the case of contraindications to spinal manipulation [8]. However, there has been limited real-world research to explore whether chiropractors use or avoid thrust-cSMT for patients with neck pain when there are potential contraindications to this therapy.

Vertebrobasilar insufficiency (VBI), also called posterior circulation insufficiency, is a condition involving transient ischemia of the posterior brain circulation often caused by vertebral or basilar artery atherosclerosis [9]. Patients with this condition may suffer from dizziness, headache, visual symptoms, and ataxia, among other symptoms [9,10], which may prompt patients to seek chiropractic care [11]. Importantly, VBI has been described as a contraindication to thrust-cSMT [8] and, more broadly, as a contraindication to any type of cervical manipulation [11,12]. A recent review (2022) included only five cases in which a chiropractor managed a patient with underlying VBI, however, thrust-cSMT was applied in three of these patients [11]. Considering VBI is described as a contraindication to cSMT yet patients with this condition have been reported to respond positively to cSMT [11], it is unclear if chiropractors typically use thrust-cSMT in the case of VBI.

Arnold-Chiari malformation describes an inferior displacement of the cerebellar tonsils through the foramen magnum and may present with varied symptoms including headache, sensory changes, vertigo, and ataxia [13]. The type of Chiari malformation (i.e., I, II, III) is classified by the extent of displacement of the cerebellar tonsils, however, patients with any type may have other clinically relevant abnormalities such as syringomyelia [14]. While some case reports have described a positive response of patients with this condition to manual therapies including cSMT [15-18], other cases have described exacerbation of symptoms following cSMT [19-22]. Given this limited and conflicting research, it is unclear if chiropractors typically use cSMT for patients with Arnold-Chiari malformation.

Anterior cervical discectomy and fusion (ACDF) is one of the most common cervical spine surgeries and is often performed for degenerative cervical spine conditions such as stenosis with myelopathy [23]. Patients with this condition may experience recurrent or persistent symptoms following surgery and present to a chiropractor for treatment [24]. However, limited research has explored chiropractors’ role in treating patients with previous ACDF, with only three published cases describing the use of cSMT after cervical spine surgery [24]. While one case reported improvements in neck pain with cSMT in a patient following ACDF, another case reported exacerbation of symptoms [25]. Considering this limited, conflicting research, it is unclear if chiropractors generally use thrust-cSMT in patients with ACDF.

Previous studies have suggested that chiropractors have different viewpoints with regard to the role of spinal manipulation and the scope of chiropractic care. For example, one recent United States survey identified three distinct subgroups of chiropractors based on their focus being: (1) spine and neuromusculoskeletal care, (2) primary care, or (3) vertebral subluxation detection and removal [26]. Studies have identified that chiropractors’ viewpoints may be influenced by or associated with their perceived role in healthcare [26-28], educational institution [29], geographic region [30], rate of diagnostic imaging [31], number of patients seen per week [31], and duration of patient encounters [28]. However, to our knowledge, the relationship between these variables and the use of cSMT in complex neck conditions remains unexplored.

Considering there are complex conditions affecting the neck which may prompt patients to seek chiropractic care, yet at the same time present a precaution or contraindication to thrust-cSMT, our primary aim was to develop and conduct a survey to explore chiropractors’ approaches to these conditions with respect to use or avoidance of thrust-cSMT. Our hypothesis was that manual thrust-cSMT would be used by less than 50% of respondents for each clinical scenario (i.e., vignette) describing a hypothetical precaution or contraindication. Secondarily, we aimed to explore if any practice characteristics independently predicted chiropractors’ choice to use thrust-cSMT.

## Materials and methods

Study design

This study was a cross-sectional survey of chiropractors in the Asia-Pacific region. The Ethics Committee of the Chiropractic Doctors Association of Hong Kong approved this study (Causeway Bay, Hong Kong; IRB ID: CDA20221031) and granted a waiver of written patient consent, as consent was implied by return of the completed survey. The survey was sent via Google Forms link to participants starting in October 2022, and the response window concluded in December 2022. The survey included details regarding consent to participate, and the respondents’ names and other identifying information were not collected so the responses remained anonymous. The study co-authors developed the survey and then conducted validity and reliability testing before disseminating it to respondents. The survey and information sheet sent to respondents are available in an open-access repository [32].

Setting and participants

The survey targeted practicing chiropractors in the Asia-Pacific region who were fluent in English, as English is an official language in several countries in this region including Australia, New Zealand, and Hong Kong Special Administrative Region. Asia-Pacific chiropractors receive their chiropractic education in a variety of chiropractic colleges internationally [33], thus potentially having different backgrounds or perspectives on the use of thrust-cSMT. The survey was sent to key organizing members of Asia-Pacific chiropractic associations, teaching institutions, and researchers, along with an information sheet describing the study. Study investigators also advertised the survey on social media.

To our knowledge, two Asia-Pacific chiropractic associations have been described in the scientific literature previously. In 2021, the Australian Chiropractors Association was reported to have over 3,000 registered chiropractors [34]. Also in 2021, there were 152 chiropractors registered within any professional chiropractic association in Hong Kong [33]. Web sources as of November 7, 2022 indicated that there are 687 registered chiropractors in New Zealand maintaining a current license [35]. The Japanese Association of Chiropractors listed 109 chiropractors as of November 9, 2022 [36]. To our knowledge, and consistent with previous research [37], other Asia-Pacific countries have fewer chiropractors. For example, a recent publication reported that India, Sri Lanka, and Indonesia have at most 10 chiropractors [37]. We did not disseminate the survey to traditional bonesetters or massage therapists such as Tui na practitioners as their practice habits would likely differ [38].

Variables

Survey items describing respondents’ practice characteristics were adapted from previous studies which included already-validated questions when possible. Chiropractors’ country of practice included a list of countries in the Asia-Pacific region with a chiropractic association. This variable was considered separately from the country of education as not all Asia-Pacific countries have chiropractic educational institutions [33]. We omitted questions regarding age and year of graduation as these could be redundant with the number of years practiced.

Multiple survey items addressed chiropractors’ educational background. Years in practice was included as chiropractic curricula have changed over time [39], thus chiropractors’ time of education could influence their perspective on cSMT use. Years in practice could also reflect providers’ confidence or experience. Country of education was derived from a list of chiropractic educational institutions provided by the World Federation of Chiropractic [40]. Hours spent reading scientific literature was also included in a previous survey [41]. We considered that practitioners aware of recent research would have a greater awareness of complex neck conditions, or alternatively could be indicative of an academic or educational role. Multiple survey items were designed to reflect respondents’ perceived role in healthcare. This included practice setting [29,30], time spent with new patients [28], rate of diagnostic imaging [31], and the view that the primary focus of chiropractic is to detect and treat vertebral subluxation [26,29].

Respondents were also asked if they would perform manual thrust-cSMT for an otherwise healthy 45-year-old female patient with localized neck pain who had no headache or neurologic deficits on exam. This question served as a control for the other clinical responses, to gauge whether a chiropractor would use cSMT in the absence of any stated complex conditions or comorbidities. Thrust-cSMT was defined in the survey as any form of spinal manipulation involving a manual impulse or thrust directed to the cervical spine.

The subsequent clinical vignettes attached a comorbidity prompt which mentioned the presence of: (1a) unilateral vertebral artery stenosis causing symptoms of posterior circulation insufficiency, including dizziness and headache, (1b) 50% stenosis of the vertebral artery unilaterally as identified by computed tomographic angiography, (2a) Arnold-Chiari malformation type I with an inferior position of the cerebellar tonsils 4 millimeters caudal to the foramen magnum, (2b) Arnold-Chiari malformation type II with an inferior position of the cerebellar tonsils 13 millimeters caudal to the foramen magnum, (3a) anterior cervical discectomy and fusion at C5/6 (i.e., using plate and screw instrumentation), performed two years previously, and (3b) anterior cervical discectomy and fusion from C3 to C6 (i.e., using plate and screw instrumentation), performed two years previously.

Respondents were asked if they would use manual thrust-cSMT for each clinical vignette, with response options including “yes,” “no,” and “don’t know/unsure.” The indeterminate response was included to allow for knowledge uncertainty, as clinicians could be unfamiliar with the question’s content or could be unable to provide a yes/no answer without additional information [42].

Data sources

The survey was the sole source of data for this study. Data were stored within the Google Forms platform and downloaded into a Microsoft Excel spreadsheet upon completion of each phase of validity testing, reliability testing, and results.

Bias

Pilot testing was used to diminish the likelihood of potential biases related to the wording of the clinical prompts. Study co-authors sent the survey to eight expert chiropractors and two expert physical therapists to determine face validity. Three of these experts also had a PhD degree. These individuals provided open-ended feedback, for example, to clarify confusing or leading questions. During piloting several changes were made to the questionnaire including omitting a question about gender, which was unnecessary to our study objectives and adding a “don’t know/unsure” response for the clinical vignettes.

To limit the likelihood that respondents would favor socially desirable responses [43], the survey included only a generic description of the research study, rather than prefacing the questionnaire by describing the prompts as “contraindications” to cSMT. The control question about the otherwise healthy patient was intended to limit bias related to differences in chiropractic approaches. Some chiropractors may typically avoid manual thrust-cSMT regardless of the patient's presentation. The each vignette includes a female of age 45, a demographic similar to the average age and demographic of chiropractic patients (mean 43.4 years, 57% female [1]). To prevent survey fatigue, the number of questions was limited to 16 items. During the piloting phase, the survey took a mean seven minutes to complete. However, the actual time to complete the final survey version was likely shorter as questions were deleted after piloting.

After pilot testing, the clinical questionnaire was disseminated to a smaller group of 38 Asia-Pacific chiropractors employed in Hong Kong and India. Considering the survey was deemed reliable (see Statistical methods), it was further disseminated without additional changes.

Study size

The sample size for reliability testing for the clinical questions was calculated using a rule of thumb estimate which requires five respondents per survey item, or in this case 35 respondents [44]. The sample size for the final version of the survey was calculated using a rule of thumb estimate for multiple logistic regression which requires 10 events per variable [45]. Considering we had 10 independent variables (covariates) in the regression model, the required minimum sample size equaled 100. This was expected to be feasible given a previous survey study administered in Hong Kong yielded 80 responses [33], and we planned to disseminate the survey to several additional regions.

Statistical methods

Descriptive statistics were calculated in Microsoft Excel while regression models were conducted using IBM Statistical Package for the Social Sciences (SPSS, Version 29.0.0.0, IBM Corp., Armonk, NY, USA). The reliability of the clinical questionnaire was examined using Guttman’s lambda six, rather than Cronbach’s alpha, as Guttman’s lambda is more applicable to smaller samples [46]. This value was calculated using a web-based software platform [47], and equaled 0.72, exceeding the minimum threshold of 0.70 for sufficient reliability [46].

A multiple binary logistic regression model was performed for each clinical vignette prompt, with the dependent variable being the decision to perform thrust-cSMT and the independent variables being the practitioner characteristics. Prior to multiple regression, we conducted bivariate correlation testing using a two-tailed Pearson correlation matrix to test for multicollinearity between covariates. Country of education and degree had a statistically significant (P<.001) correlation coefficient of 0.5, likely explained as certain countries only offer specific types of chiropractic degrees. Accordingly, the country of education was discarded from the regression models. Country of practice was also discarded as a variable, considering certain countries only had a single respondent, which led to cell counts of zero and instability of the regression model. “Don’t know / unsure” responses to the clinical vignettes were eliminated from these regression models such that the dependent variable could be analyzed as a binary response via regression.

In the regression models, the following variables were considered to represent a nominal level of measurement: responses to each clinical vignette, country of chiropractic education and practice, practice environment, level of education, and subluxation focus. The following variables were considered a scale level of measurement: years in practice, time spent with new patients, rate of imaging, and hours spent reading scientific literature.

## Results

The reporting of our results adheres to the guidelines of Strengthening the Reporting of Observational Studies in Epidemiology (STROBE) for cross-sectional studies [48].

Respondents

**Figure 1:**
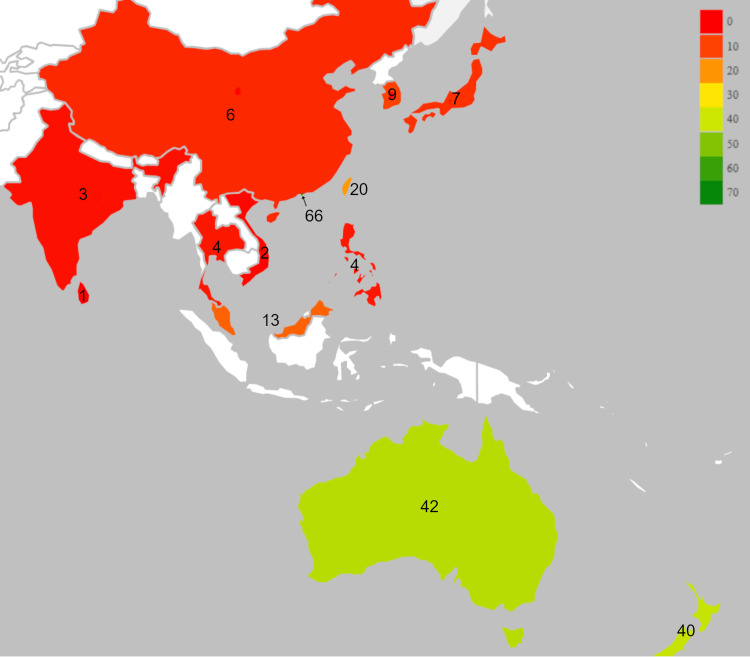
Heat map of survey respondents The number of respondents is indicated overlying the country. Countries with more participants are shaded orange, then yellow, followed by green (see legend in top right). Hong Kong Special Administrative Region is indicated by an arrow and has the most respondents (66). Image created in Microsoft Excel using the Geographic Heat Map add-in (Keyur Patel), modified by RT to include an arrow and additional shading to highlight Hong Kong Special Administrative Region and increase font size.

Respondents had on average [SD] 12.8±10.9 years in practice, spent 2.8±4.5 hours reading the scientific literature per week, and spent 38.3±17.5 minutes with new patients, and 53.4±36.8% reported obtaining imaging during the first six weeks of care. The country of education was most often the United States (34%) followed by Australia (27%), New Zealand (15%), Malaysia (11%), and other countries (13%). The country of practice was most often Hong Kong Special Administrative Region (27%) followed by Australia and New Zealand (17% each), Taiwan (8%), and other countries (30%) (Figure 1). Respondents’ highest level of education was most often a bachelor’s or double bachelor’s degree (42%) followed by a PhD or doctoral degree (32%), master’s degree (22%), diploma (3%), and other (1%). The practice setting was most often solo practitioner (33%) followed by chiropractic group practice (30%), multidisciplinary/interdisciplinary with medical physicians (23%), multidisciplinary/interdisciplinary without medical physicians (11%), and other (2%). The majority (70%) of respondents responded that the primary focus of chiropractic was to detect and treat vertebral subluxation, while 28% responded it was not, and 2% were unsure or did not know. The majority (90%) of chiropractors responded that they would use manual cervical thrust SMT on the control patient (i.e., an otherwise healthy 45-year-old woman with neck pain), while 5% stated they would not, and 5% reported they were unsure or did not know.

Use of spinal manipulation

**Figure 2:**
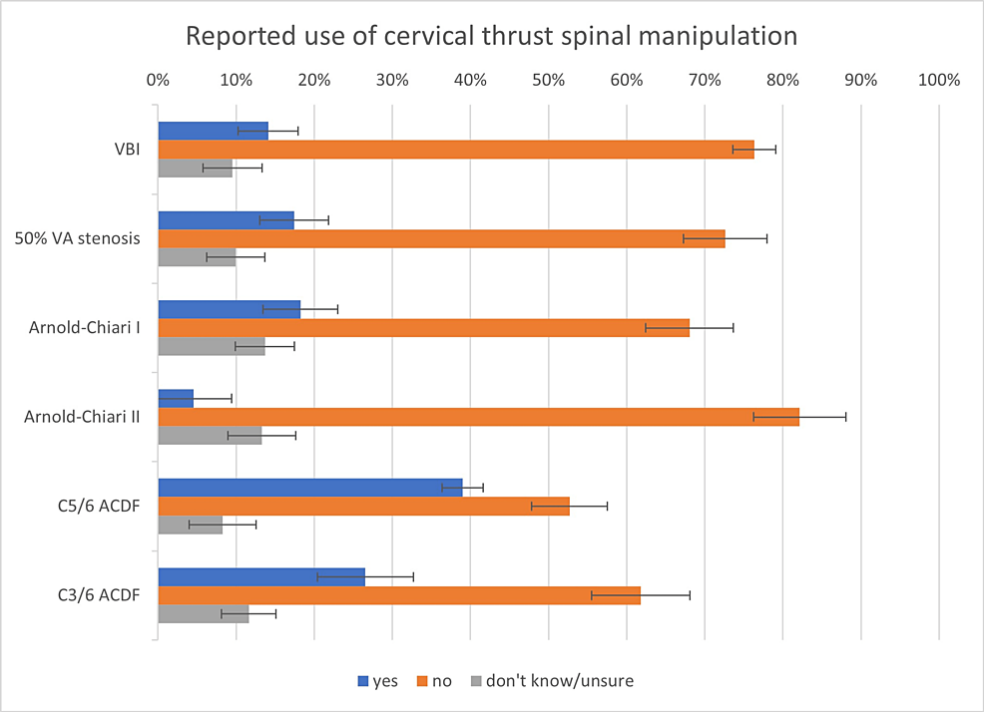
Asia-Pacific chiropractors' reported use of cervical thrust spinal manipulation in patients with complicated neck conditions ACDF: anterior cervical discectomy and fusion, VA: vertebral artery, VBI: vertebrobasilar or posterior circulation insufficiency

The percentage of chiropractors (with 95% confidence intervals [CI]) reporting the use of thrust-cSMT was less than 50% for all vignettes, including vertebral artery insufficiency (14%; 10-19%), vertebral artery stenosis (17%; 13-22%), Arnold-Chiari malformation type I (18%; 13-23%), Arnold-Chiari malformation type II (5%; 2-7%), C5/6 ACDF (39%; 33-45%), and C3-6 ACDF (27%; 21-32%). These percentages as well as the percentage of respondents indicating “no” or “don’t know/unsure” are reported along with 95% CIs in Figure 2 and Table 1.

**Table 1:** Asia-Pacific chiropractors’ reported use of thrust cervical spinal manipulation per vignette

Vignette	Yes % (95% CI)	No % (95% CI)	Don't know/unsure % (95% CI)
Vertebrobasilar insufficiency	14 (10-19)	76 (71-82)	10 (6-13)
50% stenosis of the vertebral artery	17 (13-22)	73 (67-78)	10 (6-14)
Arnold-Chiari malformation type I	18 (13-23)	68 (62-74)	14 (9-18)
Arnold-Chiari malformation type II	5 (2-7)	82 (77-87)	13 (9-18)
C5/6 anterior cervical discectomy and fusion	39 (33-45)	53 (46-59)	8 (5-12)
C3/6 anterior cervical discectomy and fusion	27 (21-32)	62 (56-68)	12 (8-16)

Regression models

Results of the regression models are shown in Table 2. The raw results, including all P-values for non-significant results, are also freely available in an online data-sharing repository [32], whereas P-values for statistically significant results (P<.05) are listed in the corresponding text below. While there were several statistically significant predictors of the use of thrust-cSMT, years in practice and rate of imaging were not significant predictors in any model. In addition, there were no significant predictors of thrust-cSMT use for the vignettes describing Arnold-Chiari malformation type II and C3-6 ACDF. Results from regression models are listed in terms of odds ratio (OR) with 95% confidence intervals [95% CI].

**Table 2:** Independent predictors of use of thrust cervical spinal manipulation per vignette An odds ratio (OR) with 95% confidence intervals [#-#] is presented for each variable. Results which yielded a significant P-value (<.05) are indicated with an asterisk (*).

	Vertebrobasilar insufficiency	50% stenosis of the vertebral artery	Arnold-Chiari malformation type I	Arnold-Chiari malformation type II	Anterior cervical discectomy and fusion C5/6	Anterior cervical discectomy and fusion C3-6
Years in practice	0.96 [0.92-1.01]	0.98 [0.94-1.02]	0.99 [0.95-1.04]	0.86 [0.76-0.98]	1.00 [1.00-1.03]	0.99 [0.96-1.03]
Greater hours reading scientific literature per week	1.16 [1.03-1.32]*	1.06 [0.96-1.17]	0.96 [0.86-1.06]	1.02 [0.81-1.29]	0.99 [0.92-1.06]	1.03 [0.96-1.11]
Fewer minutes spent with new patients	0.95 [0.92-0.99]*	0.96 [0.93-0.99]*	0.95 [0.92-0.98]*	0.98 [0.93-1.02]	0.99 [0.97-1.01]	0.99 [0.96-1.01]
Subluxation focus (reference: no)	5.89 [1.56-22.26]*	2.16 [0.78-5.99]	1.19 [0.46-3.05]	5E8 [0-infinite]	1.67 [0.82-3.38]	2.05 [0.94-4.49]
Bachelor’s or double bachelor’s degree (reference: diploma)	0.50 [0.04-6.19]	0.21 [0.03-1.67]	8E7 [0-infinite]	6E6 [0-infinite]	0.59 [0.08-4.41]	0.78 [0.07-8.30]
Master’s degree (reference: diploma)	0.10 [0.01-1.89]	0.04 [0.00-0.53]*	2E8 [0-infinite]	2E7 [0-infinite]	1.17 [0.15-8.98]	1.55 [0.14-16.69]
PhD / doctoral degree (reference: diploma)	1.03 [0.09-11.95]	0.45 [0.06-3.30]	4E8 [0-infinite]	3E7 [0-infinite]	1.39 [0.19-10.21]	1.98 [0.19-20.43]
Group practice (reference: solo practice)	0.53 [0.18-1.58]	0.33 [0.11-0.97]*	0.48 [0.16-1.45]	0.28 [0.05-1.75]	0.53 [0.24-1.14]	0.62 [0.26-1.46]
Multidisciplinary/interdisciplinary practice with medical physicians (reference: solo practice)	0.26 [0.07-1.02]	0.44 [0.14-1.43]	0.56 [0.17-1.85]	0.17 [0.02-1.51]	0.62 [0.26-1.47]	0.52 [0.19-1.42]
Multidisciplinary/interdisciplinary practice without medical physicians (reference: solo practice)	0.58 [0.13-2.60]	0.76 [0.21-2.76]	0.88 [0.22-3.5]	0.00 [0-infinite]	0.62 [0.23-1.68]	1.05 [0.35-3.16]
Use of manual cervical thrust manipulation on a healthy patient (reference: no)	2.75 [0.26-29.06]	3.94 [0.39-40.42]	4E8 [0-infinite]	7E7 [0-infinite]	12.36 [1.49-102.66]*	6.97 [0.84-58.14]
Greater rate of imaging	0.99 [0.98-1.00]	1.00 [0.99-1.01]	1.01 [0.99-1.02]	1.00 [0.98-1.02]	1.00 [0.99-1.01]	1.00 [0.99-1.01]

For the vignette describing VBI, greater hours per week reading scientific literature was associated with an increased likelihood of reported use of thrust-cSMT (OR 1.16 [1.03-1.32], P=.016). Fewer minutes spent with new patients was associated with a reduced likelihood of reporting thrust-cSMT (OR 0.95 [0.92-0.99], P=.006). Chiropractors who agreed that the primary focus of chiropractic is to detect and treat vertebral subluxation were more likely to report the use of thrust-cSMT (OR 5.89 [1.56-22.26], P=.009).

For the vignette describing 50% stenosis of the vertebral artery, chiropractors with a master’s degree were less likely to report the use of thrust-cSMT (OR 0.04 [0.00-0.53], P=.014). Chiropractors in a chiropractic group practice were also less likely to report the use of thrust-cSMT (OR 0.33 [0.11-0.97], P=.043). Decreased minutes spent with new patients was associated with a reduced likelihood of reporting the use of thrust-cSMT (OR 0.96 [0.93-0.99], P=.006).

For the vignette describing Arnold-Chiari malformation type I, reduced minutes spent with new patients was associated with a reduced likelihood of reporting the use of thrust-cSMT (OR 0.95 [0.92-0.98], P<.001). For the vignette describing C5/6 ACDF, chiropractors reporting the use of thrust-cSMT on the healthy control patient were also more likely to report the use of thrust-cSMT in this scenario (OR 12.36 [1.49-102.66], P=.020).

## Discussion

This cross-sectional survey of Asia-Pacific chiropractors was the first study to our knowledge that explored chiropractors’ use of cSMT in patients with neck pain and precautions or contraindications to this treatment. Our findings supported our hypothesis as greater than half of the respondents indicated that they would not use thrust-cSMT in each clinical vignette of precaution/contraindication, suggesting that chiropractors are cautious with the use of thrust-CSMT in these scenarios. Further, we identified several significant independent predictors of the use of thrust-cSMT. The most frequent predictor was reduced minutes spent with new patients, which predicted a reduced likelihood of thrust-cSMT in three vignettes. Other predictors were significant in one vignette each including a focus on subluxation, degree, practice environment, use of thrust-cSMT on a healthy patient, and hours reading scientific literature.

To our knowledge, there are few survey studies that have examined chiropractors’ use of thrust spinal manipulation in cases of precaution or contraindication to this therapy. In one study of United States’ chiropractors, 54% of respondents indicated that they would either “rarely” or “never” use thrust manipulation in the lumbar spine of a patient with a surgical lumbar fusion [49]. Our study likewise showed that most Asia-Pacific chiropractic respondents indicated they would avoid thrust-cSMT in the cervical spine in the presence of a surgical fusion (i.e., ACDF).

One previous survey study of Australasian chiropractors found that only 38% would use thrust-cSMT for patients with cervical spinal stenosis [2]. While stenosis is typically what prompts ACDF, underlying stenosis was not specifically queried in the current study. However, our results suggested that only a minority of chiropractors would use thrust-cSMT in patients with ACDF (39% and 27% per each vignette). Our results could therefore be consistent with a rationale to avoid thrust-cSMT in the presence of current or previous cervical spinal stenosis regardless of a history of surgery.

We are unaware of any survey studies that examined chiropractors’ use of cSMT in patients with vertebral artery disorders or Arnold-Chiari syndrome. Until now, the use of cSMT in these patients could only be ascertained from case reports, which may have an inherent publication bias. To our knowledge, there are only five published cases in which a chiropractor managed a patient with VBI, and thrust-cSMT was applied in three of these patients [11]. Likewise, we are only aware of case reports describing the use of cSMT for patients with Arnold-Chiari syndrome with outcomes being positive [15-18], or negative [19-22]. Our study suggests that Asia-Pacific chiropractors tend to avoid thrust-CSMT in the presence of disorders of the vertebral artery and Arnold-Chiari malformation.

While the most frequent predictor of avoiding thrust-cSMT was a reduced number of minutes spent with new patients, it is unclear why this was the case. New patient examinations often consist of history taking and physical examination procedures to characterize the source of the patient’s chief complaint. Therefore, it is possible that chiropractors who spend less time during examination have less information to base their treatment and are accordingly more cautious. Further research is needed to explore factors related to thrust-cSMT use and time spent with patients.

While most respondents indicated they would use thrust-cSMT on a healthy patient (i.e., 90%), a minority indicated they would not, or were unsure. We suspect that the reason for this finding is that certain chiropractors tend to avoid thrust-cSMT altogether. There is a range of techniques of spinal manipulation, some of which do not involve a manual thrust. One of the most common non-thrust techniques is instrument-assisted manipulation (i.e., Activator® technique). In a survey of British chiropractors, 2% of respondents indicated they typically used this method. In another survey of Australasian chiropractors 36% indicated they would use instrument-assisted manipulation on a patient with myofascial pain (e.g., without precautions/contraindications) [2].

A master’s degree (compared to a diploma) independently predicted a reduced likelihood of use of thrust-cSMT in the vignette describing 50% stenosis of the vertebral artery. While it is unclear why educational level impacted thrust-cSMT use in this vignette, we suggest that different educational curricula may lead to different practice behaviors. Chiropractic educational requirements and scope of practice vary across the Asia-Pacific region, however, any individual completing a formal chiropractic educational program is referred to as a “chiropractor.” One recent survey of the chiropractic profession in Australia, which has its own chiropractic educational institutions, reported that most chiropractors had a bachelor’s degree (35%) followed by master’s (33%) and doctoral (30%) degree, with the remaining percentage having a diploma [50]. In contrast, in Hong Kong Special Administrative Region, where most chiropractors are educated in other countries, 83% reported having a doctoral degree [33].

The chiropractic profession is officially recognized and regulated in several Asia-Pacific countries, including Australia, Hong Kong Special Administrative Region, Japan, Malaysia, New Zealand, The Philippines, Singapore, and Thailand [51]. The legal status of chiropractic is unclear in China, India, South Korea, Taiwan, and Vietnam [51]. Individuals in countries where the legal status of chiropractic is unclear may obtain a diploma in chiropractic technique after undergoing a brief one-month course [52], and could refer to themselves as chiropractors in these countries. However, we accounted for this by disseminating our survey to chiropractors who completed a formal chiropractic degree program. Accordingly, we aimed to exclude chiropractors who only received a brief diploma. However, chiropractic degree offerings have changed over time, and chiropractic colleges in the Asia-Pacific region previously offered chiropractic graduate diplomas which required one or two years of education [53].

In the present study, only a small percentage of chiropractors received a chiropractic diploma (3%), all of which were obtained in a country where chiropractic is legally regulated. These degrees were therefore likely formal graduate diplomas. This also suggests that the present study did not include respondents with a diploma obtained through an abbreviated program in a country where chiropractic is not regulated. The duration of study required to obtain a bachelor’s, master’s, or doctoral degree of chiropractic varies per country and program. Beyond the diploma level, chiropractic programs typically require a minimum of four to five years of education [54], in addition to any undergraduate prerequisites, which may include an additional three years of education [55].

These results may inform future studies including prospective cohort studies involving the treatment of patients with these conditions. For example, the clinical scenarios listed (VBI, Arnold-Chiari malformation, ACDF) could be listed as exclusions in the eligibility criteria of experimental study designs, as chiropractors tend to avoid thrust-cSMT in these patients.

This study has certain limitations. Not all Asia-Pacific chiropractors are fluent in English, which effectively reduced the available sample size. While the sample size was sufficient for our regression models, certain countries were poorly represented in relation to the number of practicing chiropractors. The clinical vignettes may be oversimplified, whereas in a real-world setting chiropractors could perform additional examination procedures, order further testing, or consult with a medical specialist before deciding to use thrust-cSMT. As Arnold-Chiari malformation type I may be identified incidentally via imaging, the source of the patient’s neck pain or reason for chiropractic treatment in this vignette could have been made clearer. As a survey study, respondents may opt to provide socially desirable responses [43]. This could manifest as respondents tending to state they would avoid thrust-cSMT if they suspected our research team would prefer to see this response. Although respondents remained anonymous, reporting the use of thrust-cSMT in a potentially contraindicated disorder may be perceived as sensitive information, and respondents may be unwilling to disclose their practice habits [43]. The results of this survey are only generalizable to the Asia-Pacific region, as different regions of practice could influence chiropractors’ responses. Our findings do not apply to practitioners who completed a brief diploma in chiropractic techniques in a country where chiropractic is unregulated by law. We were unable to determine the number of chiropractors who received the questionnaire and therefore could not calculate a response rate. Due to having several predictor variables, certain cells had low counts during regression, leading to infinite confidence intervals for certain outcomes. Our regression models may have also been confounded by unmeasured variables such as provider gender/sex, previous adverse events, malpractice claims, or concurrent use of other therapies (e.g., soft tissue manipulation, exercise).

Although this study identified key predictors of cSMT use, it cannot answer the question of which conditions represent an absolute contraindication to cSMT, or which cSMT approaches would lead to improved clinical outcomes with regard to neck pain or other outcomes. Further studies regarding the safety and effectiveness of cSMT in these patient populations are needed. However, as the clinical presentations described in our survey may be uncommon and could represent contraindications to cSMT, such research may be challenging or unethical to conduct.

Follow-up surveys also could examine each of the conditions of precaution/contraindication in greater depth. An individual survey for each condition (i.e., vertebral artery disorders, Arnold-Chiari malformation, ACDF) would allow for more detailed questions about spinal manipulation technique selection. For example, it is not only important to know if thrust-cSMT is avoided, but what treatments are provided in place of this treatment (i.e., exercise, soft tissue therapy, no therapy, etc.). Further research is also needed to explore the influence of several other risk factors on chiropractors’ decision to use cSMT, such as hypertension, osteoporosis, connective tissue disorders, and smoking [12]. In addition, the current and follow-up surveys could be administered to chiropractors in other regions of the world.

## Conclusions

This study was the first to explore chiropractors’ use of thrust-cSMT for complicated neck pain and identified that Asia-Pacific chiropractors typically avoid this treatment in the presence of potential contraindications including vertebral artery disorders, Arnold-Chiari malformation, and ACDF. Chiropractors’ use of thrust-cSMT in the presence of complicated neck pain may depend on providers’ characteristics, including time spent with new patients, focus on subluxation, degree, group practice environment, use of thrust-cSMT on a healthy patient, and weekly hours reading scientific literature. Further research is needed to explore reasons why chiropractors use or avoid thrust-cSMT in specific clinical scenarios.
